# Summer Success: SKIPing to Motor Competence for Disadvantaged Preschoolers

**DOI:** 10.3390/children12050578

**Published:** 2025-04-29

**Authors:** Dimetrius Brandon, Ruri Famelia, E. Kipling Webster, Jacqueline D. Goodway

**Affiliations:** 1 Department of Kinesiology, Recreation and Sport Studies, University of Tennessee, Knoxville, Knoxville, TN 37996, USA; dbrando6@vols.utk.edu (D.B.); kipwebster@utk.edu (E.K.W.); 2 Center for Biobehavioral Health, Abigail Wexner Research Institute, Nationwide Children’s Hospital, Columbus, OH 43205, USA; ruri.famelia@nationwidechildrens.org; 3 Department of Kinesiology, Michigan State University, East Lansing, MI 48824, USA

**Keywords:** fundamental motor skills, intervention, disadvantaged preschoolers

## Abstract

**Background**: Disadvantaged children often enter kindergarten with delays in fundamental motor skill (FMS) competence, which is critical for future physical activity engagement. The Summer Success—Successful Kinesthetic Instruction for Preschoolers (SS-SKIP) program was designed to address these developmental gaps, with a short, intensive intervention. This pilot study evaluated the impact of a 4-week SS-SKIP program on FMS, perceived motor competence (PMC), and executive function (EF). **Methods**: Twenty-one preschool children (mean age = 62.62 ± 4.61 months) from disadvantaged communities participated in an intensive, month-long (240 min) program. FMS were assessed using the Test of Gross Motor Development-2 (TGMD-2), PMC was evaluated using the Pictorial Scale for Perceived Competence, and EF was measured via the Head–Toes–Knees–Shoulders (HTKS), Go/No-Go, and Sorting cards tests. Standing long jump was measured in meters. A pretest–post-test design assessed program impact using 2 Gender X 2 Session MANOVAs/ANOVAs on dependent variables. **Results**: Analysis of differences in baseline measures of FMS competence and EF by Gender and Session revealed no significant main effects of Gender, Session, or their interaction across measures (all *p* > 0.05). Repeated measures ANOVAs by Gender revealed a significant main effect for Time for locomotor standard scores (*p* < 0.001), object control standard scores (*p* < 0.001), and HTKS scores (*p* < 0.001), indicating improvement from pretest to post-test. By contrast, jump distance, PMC, Go/No-Go and Card Sorting scores were non-significant (*p* > 0.05). **Conclusions**: A short, intense SS-SKIP FMS intervention significantly enhanced FMS and improved HTKS performance. This pilot study was limited by the lack of a control group and small N. These findings underscore the potential of short, targeted interventions in addressing early motor delays in disadvantaged preschoolers, warranting further investigation into their long-term impacts.

## 1. Introduction

Many young children from disadvantaged environments (i.e., low socioeconomic households) enter kindergarten without the prerequisite skills to be successful in school, setting them on a negative educational trajectory [1]. There are significant inequities for disadvantaged children from low-socioeconomic backgrounds who are not as prepared for formal schooling as their peers from higher-income backgrounds [2]. These inequalities stem from inconsistencies in pre-kindergarten education experiences, lack of resources (e.g., books, educational games) in the home environment, and consequently limited parental involvement in the cognitive and social development of their child [3,4]. Many of these children have had no access to organized childcare, have had little/no exposure to organized sports, and have been raised in low-income homes by parents with lower educational attainment [5,6]. In addition to concerns about academic readiness, research shows that children from disadvantaged environments also demonstrate developmental delays in critical motor skills that are prerequisites for sport-specific skills [7]. Early motor skill interventions are warranted, as these children have been identified as at-risk of demonstrating developmental delays in their motor skills in preschool years, which set them on a negative developmental trajectory for physical activity [8,9,10,11].

Fundamental motor skills (FMS) are considered to be the “ABCs” of motor skills and movement patterns [12] and include locomotor skills such as running, jumping, and hopping and object control skills such as throwing, kicking, and catching. These FMS are building blocks to acquire more advanced movements required to participate in physical activity, sports, and games [12,13,14,15]. It has been typically found that gender differences exist in object control skills, but this is less true for locomotor skills [6]. A common misconception is that FMS naturally emerge; however, these skills must be taught, practiced, and reinforced over time [16]. Studies show that children from socially disadvantaged environments face developmental delays and challenges in their development of FMS [8]. Additionally, mastery of FMS contributes to a child’s physical, cognitive, and social development [17] and is important in the overall development of critical cognitive skills such as executive function (EF) [18].

One highly cited model of motor development by Stodden et al. [19] suggests that FMS competence is an underlying mechanism driving physical activity (PA) behaviors across the lifespan. Thus, when children have low FMS competence, they get drawn into a negative spiral of disengagement from PA and are at a greater risk of developing obesity and having a greater likelihood of risk for cardiovascular diseases [20]. Thus, it is particularly concerning that many young children from disadvantaged environments demonstrate significant delays in their FMS competence and are in need of intervention [8,9,21,22].

In addition to the vital role FMS competence plays in overall health [22,23], the Stodden et al. model also highlights the importance of perceptions of motor competence [13] (PMC). A child’s perceived motor competence refers to the way in which a child views or thinks of her/his competence in movement situations [19]. Harter and Pike [24] suggest that, in the early and middle childhood years, perceived motor competence (mostly in play environments) is the most important. By later childhood, perceived athletic competence emerges and is more focused on movement in sports and games [24]. Children who perceive themselves as highly competent are more inclined to persist in challenging movement tasks [24]. Additionally, a strong sense of PMC can enhance children’s motivation to improve their motor skills and engage in PA [25]. However, previous studies have found that young children often have an inflated PMC [26,27]. In the short term, as children overestimate their actual motor competence, it can serve as a positive motivator to increase their PA, but in the long term a mis-match between actual motor competence and PMC can be detrimental to engagement in PA [20]. Thus, both PMC and FMS competence play a significant role in a child’s motivation to learn and engage in current and future FMS and PA [28].

There is also significant concern about school readiness skills and EF, along with FMS development of children from disadvantaged environments, necessitating the need for early intervention. EF skills are particularly important to develop in early childhood. EF refers to a set of cognitive processes that enable a child to engage in goal-directed behavior by regulating their thoughts, emotions, and actions [29,30,31]. These skills are critical for problem-solving, decision-making, and self-control. There are three core components of EF: (1) working memory (ability to hold and manipulate information in the brain), (2) inhibitory control (the ability to resist impulses and distractions), and (3) cognitive flexibility (the ability to switch between tasks or think about multiple things at once in a problem-solving environment). EF skills are essential to academic success and emotional regulation and should be worked on during the preschool years [29,30]. Thus, not only is it important to develop FMS during the preschool years, but it is also important to develop EF [32,33,34]. Due to the limited instructional time available in early childhood, interventions that could develop the two simultaneously would be particularly valuable [35].

One well-cited early childhood FMS intervention is called Successful Kinesthetic Instruction for Preschoolers (SKIP), and was developed by Goodway and colleagues (see Goodway et al. [6] for a detailed description of the program). The SKIP program has proven to be successful in significantly improving FMS and PMC in children from disadvantaged backgrounds at various dosages from 6 to 12 weeks [6], with effect sizes ranging between η^2^ = 0.55 and η^2^ = 0.70. In fact, a SKIP intervention as short as 6 weeks was able to yield an effect size of η^2^ = 0.63 [36]. However, what is not clear from the literature is whether a short, intense FMS intervention can yield the same effect sizes as longer SKIP interventions. This pilot study was unique to other SKIP studies in that it had a short, intense duration and was set within an innovative summer program called Summer Success (SS). SS was designed to ensure preschool-aged children from low-income backgrounds had a successful transition into kindergarten [37]. The program emphasizes the development of language, literacy, math, social–emotional, and motor skills for preschoolers who had received limited formal early learning experiences. This initial study was a proof of concept that tested the efficacy of a short, intense SS-SKIP program for children who had never received formal preschool on the FMS of the children.

The overall purpose of this pilot study was to evaluate if a 4-week pilot FMS intervention, Summer Success—Successful Kinesthetic Instruction for Preschoolers (SS-SKIP), delivered as part of the overall Summer Success school readiness program would significantly improve FMS in preschool-age children about to enter kindergarten. It was hypothesized that children who participated in the SS-SKIP program would significantly improve their FMS from pretest to post-test. Additionally, secondary hypotheses investigated whether there were gender differences in baseline locomotor and object control skills, and whether participation in SS-SKIP would lead to significant improvements in the secondary outcomes of PMC and EF.

## 2. Methods

### 2.1. Recruitment and Participants

This study was conducted at a university-affiliated childcare center situated in a Midwestern city in the United States. Children were recruited through fliers and information sessions at neighborhood elementary schools, a local home-visiting program, and a local public housing program. Inclusion criteria for child participants included the following: children from low-income backgrounds; entering kindergarten in the fall; and with little-to-no formal early childhood education experience prior to kindergarten. Exclusion criteria included any child with a documented developmental disability. The majority of participants were beneficiaries of public assistance programs such as the Women, Infants, and Children (WIC) or the Supplemental Nutrition Assistance Program (SNAP), received free or reduced lunch, or lived in public housing. Given the nature of this proof-of-concept pilot study, all participants received the SS-SKIP programming. Thus, a limitation of this study was the lack of a control group. A total of 21 children (10 boys and 11 girls; Mean age = 62.62 ± 4.61 months) were enrolled in the SS-SKIP intervention (see Table 1). The majority of participants identified as African American (61.9%), followed by Multiracial (19.0%), Hispanic (14.3%), and White (4.8%).

### 2.2. Design and Variables

This study used a pretest–post-test design to evaluate the SS-SKIP intervention. The primary dependent variable for this study was FMS. In addition, PMC and EF (including inhibitory control, working memory, and cognitive flexibility) were examined as exploratory outcomes. Furthermore, height and weight were measured to calculate body mass index (BMI). The independent variable for the participants was the four-week SS-SKIP intervention.

### 2.3. Measures

*FMS Competence: Test of Gross Motor Development 2nd Edition (TGMD-2)*. The TGMD-2 is a validated, norm- and criterion-referenced assessment that was used to measure FMS in children [38]. This assessment included two subscales with six skills (run, gallop, hop, leap, horizontal jump, slide) making up the locomotor subscale and six skills (two hand strikes, stationary dribble, catch, kick, overhand throw, underhand roll) comprising the object control subscale. The participants were digitally recorded performing three attempts of each skill with the first attempt being a practice trial while the second and third were scored independently by trained coders for this study. Each skill was evaluated on 3 to 5 performance criteria such as “hands in front of body” for catching. Performance criteria were evaluated for their presence or absence during each trial. If a child demonstrated the performance criterion, they received 1 point; if not, they received a 0. The total raw scores ranged from 0 points to 48 points for both locomotor and object control subscales. Each subscale was converted into standard scores and percentile ranks using age- and sex-specific normative values [38]. TGMD-2 is a highly reliable assessment of FMS in children, demonstrating strong internal consistency (Cronbach’s alpha > 0.9), and robust test–retest reliability (r = 0.84–0.96), making it a dependable tool for evaluating FMS [38].

Prior to this study, research staff responsible for coding the FMS data went through multiple training sessions with two TGMD-2 experts and had to reach an initial inter-observer agreement of at least 95% reliability against the experts prior to the start of the coding to ensure inter-rater (between coders) reliability among coders.

*FMS Competence: Standing Long Jump*. Children performed three trials of a standing long jump, and the distance in meters was measured. Children started with their feet behind a line, and the distance jumped from take-off to the closest heel on landing was measured. The longest jump was recorded for analysis.

*Perceived Motor Competence: Pictorial Scale for Perceived Competence and Social Acceptance*. The PMC of the participants was assessed using the physical competence subscale of the Pictorial Scale for Perceived Competence and Social Acceptance [24]. This subscale consists of six items featuring gender-specific images of boys or girls engaging in different physical activities. The skills in the scale included running, skipping, hopping, tying shoelaces, climbing, and swinging. Each skill is depicted by presenting two pictures side by side: one showing a highly skilled child and the other a less skilled child. Children completed a double, dichotomous response to indicate which picture they were “most like” (high skill/low skill) and then whether they were “a lot” or “a little” like that image to reflect a score ranging from 1 to 4. A score of 4 indicated that the child perceived themselves as highly skilled, and a score of 1 indicated low PMC. The mean score across these six items was used in the analysis. The scale has demonstrated good reliability, with test–retest correlations ranging from 0.80 to 0.92, and strong validity [24].

*Executive Function: Head–Toes–Knees–Shoulders (HTKS)*. The HTKS assessment is a validated and reliable tool for evaluating behavioral self-regulation in preschool-aged children [39]. This assessment involves participants responding to verbal commands by performing actions that are opposite to those instructed, demonstrating their ability to inhibit dominant responses. For instance, if asked to “touch your toes,” the correct response would be to touch their head, and vice versa. The HTKS assessment is structured into three parts, each consisting of verbal instructions, 4–6 practice trials, and 10 test trials. Participants are scored based on their performance—receiving a score of 2 for completing a trial correctly, 1 for self-correcting during a trial, and 0 for an incorrect response. To proceed to the next stage of the assessment, participants must have achieved a minimum score of 4 points during the previous test trials. In the second part of the assessment, participants incorporate additional commands involving their knees and shoulders, altering the complexity of the task. The final part further modifies the rules, requiring participants to associate head with knees and toes with shoulders. Trained administrators live coded the predetermined trials. The score was calculated by summing the points earned during the test trials, resulting in a total score ranging from 0 to 60. A higher score indicated better behavior regulation and EF in preschool children. The HTKS demonstrated strong reliability, with internal consistency coefficients (Cronbach’s alpha) ranging from 0.85 to 0.93 and test–retest reliability correlations of 0.74 to 0.92 across diverse samples of young children [39].

*Executive Function: Go/No-Go, Early Years Toolbox (EYT)*. The Go/No-Go task from the Early Years Toolbox (EYT) is an iPad-based assessment designed to evaluate inhibitory control, specifically the ability to manage behavioral impulses [40]. In this task, children are shown fish and sharks on the screen. They are instructed to tap the screen when a fish appears (“catch the fish”—Go stimulus) and to avoid tapping when a shark appears (“avoid the sharks”—No-Go stimulus). The task begins with two sets of five practice trials: one set where only fish appear and one set where sharks are included as No-Go stimuli. Following the practice, children complete three rounds of 25 trials, with fish appearing in 80% of the trials (Go) and sharks in 20% (No-Go). Overall scores were calculated by summing the correct “Go” responses and correct “No-Go” inhibitions, then multiplying the total by 100 to determine the overall accuracy. The Go/No-Go demonstrated good reliability, with test–retest reliability coefficients ranging from 0.5 to 0.85 across preschool children [40,41].

*Executive Function: Card Sort Task, EYT*. The Card Sort Task from the EYT is an iPad assessment designed to evaluate cognitive flexibility by asking children to sort cards (e.g., red rabbits, blue boats) according to two different sorting dimensions: color and shape. The task begins with a demonstration trial and two practice trials, after which children complete six test trials where they sort the cards by one dimension (e.g., color). Following these trials, the children enter a post-switch phase, where they are instructed to sort the same cards by the alternate dimension (e.g., shape). Before each trial, the relevant sorting rule is reiterated, and a stimulus card is presented for sorting. To progress to the next round, children must correctly sort at least five out of the six stimuli in both the pre-switch and post-switch phases. Participants who meet this criterion move on to a “border phase”. In this phase, children are required to sort cards by color if the card has a black border or by shape if the card lacks a border. This phase also begins with a demonstration and two practice trials, followed by six test trials, which include three bordered and three non-bordered stimuli. Throughout all phases of the task, no particular stimulus is presented more than twice consecutively. Scoring is based on the number of correct sorts after the pre-switch phase. The Card Sort Task has shown good reliability, with test–retest reliability coefficients ranging from 0.78 to 0.87 [41].

### 2.4. Summer Success Program

Given the importance of promoting school readiness skills, and FMS development, an innovative, intense, month-long summer program called Summer Success was developed to better prepare preschool children from disadvantaged environments for entry into kindergarten. The goal of Summer Success was to promote a broad range of readiness skills (reading/math/socio-emotional skills) including FMS development for disadvantaged preschoolers who had no prior schooling experience and were preparing to enter kindergarten in the fall following the summer programming. The Summer Success program was designed to be a short, intensive, four-week program based on professional practices and programs supported by empirical evidence in literacy, math, and emotional development. This evidence-based program was designed by researchers in partnership with parents and educators who believed these skills were important for success in kindergarten. Khan et al. (2017) provides more detail on Summer Success [37]. SS-SKIP was situated within the overall Summer Success program.

Full-day and half-day sessions of Summer Success were implemented in the first year to evaluate caregivers’ interest in full- versus half-day programming and also to examine corresponding outcomes relative to the dose of the intervention. The full-day Summer Success program took place in June (*n* = 11) and consisted of 7 h of programming per day and 35 h per week for four weeks. The half-day program in July (*n* = 10) consisted of 3 h of programming per day and 15 h per week for four weeks.

### 2.5. SS-SKIP Intervention

The SS-SKIP program was founded and adapted from the SKIP FMS intervention for young children [5]. The intervention was run by a motor development expert with a doctoral degree in Kinesiology, Physical Education. The expert had extensive experience running FMS programs and interventions for children with developmental delays. Two undergraduate researchers who were obtaining Kinesiology undergraduate degrees assisted in running the intervention. The assistants had taken a course in motor development and previously worked with children with developmental delays.

### 2.6. Theoretical Foundation of SS-SKIP

The SS-SKIP intervention was grounded in Dynamical Systems Theory (DST) using Newell’s constraints perspective [42]. The DST purports that movement is a product of the interaction between constraints from the child, task, and environment [42] thus no two children follow the same developmental trajectory. An evidence-based three-step approach to the design of SS-SKIP instructional activities was utilized (see [6] for a more detailed description of the SKIP program).

The first core premise in SKIP is that all instruction starts from the child. Individual constraints that influenced children’s ability to perform a skill (e.g., lack of tracking a ball, poor dynamic balance) were identified along with the stage of development the children demonstrated for each skill based off the TGMD-2 baseline. For example, a child who had poor tracking abilities might demonstrate a catch where they hugged the ball [6]. The second core premise of SKIP was modification of environmental factors (equipment, environment) to align with the developmental level of the child. Equipment factors such as ball size, shape, texture, and other physical prompts such as poly spots to step on or targets on the wall were considered and manipulated to align with the child’s development. For example, if a child caught a ball by a “hugging” it, a large foam ball might be selected for them to catch so they had a better chance at catching the ball with just their hands. The third core premise of SKIP was the design of developmentally appropriate tasks for each child’s developmental stage. It was ensured that the tasks (1) were motivating so the child felt compelled to participate, (2) were aligned with the child’s stage of development, and (3) involved maximum opportunities to respond to the task. For example, for the same child who hugs while catching, a large light ball tossed from a short distance with increasing distance as the child successfully caught it (“catch the coconuts across the crocodile-infested river”), might be selected. Lastly, the tasks were regularly modified according to the child’s current performance. To enhance the child’s performance, the child received specific instructional and positive feedback and regular modeling (demonstrations).

### 2.7. Organization of SS-SKIP

The SS-SKIP program was developed to be a short intervention aimed at improving FMS in preschool children. Two identical SS-SKIP programs were implemented at a university-affiliated childcare center (half-day, full-day). Each program consisted of eight sessions over the 4 weeks, with each session lasting 30 min, for a total instructional dose of 240 min. Both programs followed the same curriculum, focusing on a variety of developmentally appropriate locomotor and object control activities. Each session began with a 3-min warmup, led by the undergraduate assistants, which included music, with three locomotor skills being taught and practiced. The participants worked on locomotor skills such as run, gallop, hop, skip, jump, leap, and slide. As locomotor skills typically develop earlier than object control skills [6], less emphasis was placed on these skills than on object control skills.

Following the locomotor warmup, the participants were divided into two different groups where they practiced object control skills. Object control skills are more ontogenetic and take longer to acquire; thus, in a short program like SS-SKIP [6], it was decided to prioritize these object control skills. Each session, the instructors led two-to-three object control skill stations where they explained and demonstrated the tasks to the participants. Children engaged in their station for 12 min, receiving feedback, refinements, and extensions from their instructor at their station. After the skill stations were completed (24 min total), the participants were gathered in a circle with the instructors. The instructors spent the last 3 min of the lesson conducting a cool-down, debriefing, and giving children feedback on the different locomotor and object control skills they worked on. The lead and assistant researcher ensured that all sessions adhered to this format. The lead researcher had been trained extensively in delivering SKIP and had delivered SKIP interventions for over three years. She had previously been evaluated as delivering SKIP against the designer of SKIP to a reliability of over 97%.

### 2.8. Procedures

All study procedures were approved by a university institutional review board and the childcare center that granted permission to conduct this study. Written parental permission was secured from the parent/legal guardian of the child prior to participation. Verbal assent was also provided by the child participating in the study.

Participants completed the pretest during the first week of the intervention on day one using the TGMD-2 [38] to assess baseline object control and locomotor skills. Jump distance was also recorded during the jump trials of TGMD-2. On day one, during classroom time, children were also pretested on their PMC, EF (HTKS, Card Sorting, Go/No-Go), and anthropometrics. After completing the SS-SKIP program, participants underwent post-test assessments on day eight following the pretest measures.

### 2.9. Statistical Analysis

Descriptive statistics were conducted on the FMS and exploratory outcomes, including PMC and EF. Two separate MANOVAs by Gender and Session on (1) pretest locomotor and pretest object control standard scores (SSs) and (2) HTKS raw scores, Go/No-Go scores, and Card Sorting scores were conducted to look at pretest differences by Gender and Session. Two separate univariate ANOVAs by Gender and Session on (1) jump distance and (2) PMC were conducted to look at pretest differences in these variables. In order to investigate the influence of the 4-week SS-SKIP program, univariate ANOVAs with repeated measures by Gender were conducted on locomotor SSs, object control SSs, and long jump, PMC, HTKS, Go/No-Go and Card Sort scores. Session was not included in the analysis as it was not deemed to be significant in the baseline analyses. We determined the eta squared effect size using Cohen’s (1998) thresholds for interpretation, defining small, medium, and large effects as 0.01, 0.06, and 0.14, respectively [43].

## 3. Results

### 3.1. Baseline

The sample consisted of 21 participants (10 boys, 11 girls) with a mean BMI percentile of 47.05 (*SD* = 36.10), demonstrating that most children fell within the normal weight range. All participants received the SS-SKIP pilot intervention, with 11 completing session one (i.e., June) and 10 completing session two (i.e., July). Three participants were not there on the last days of data collection for our final measures—two from session one and one from session two (see Table 2).

### 3.2. Baseline Measures of FMS, PMC, and EF

**Figure 1:**
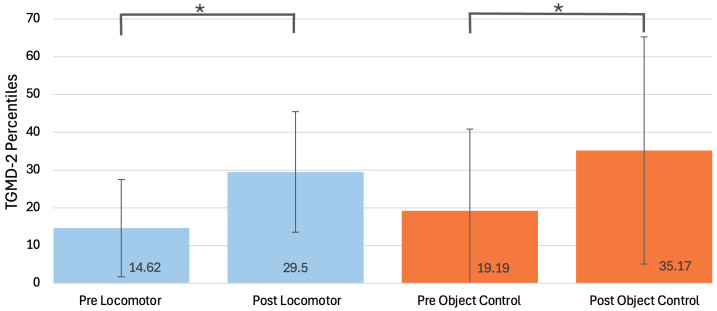
Pre and post TGMD-2 percentile. Note: * indicates significant results (*p* < 0.001).

Due to the small sample and cell size, Gender and Session differences were run separately with an adjusted alpha of *p* = 0.0125 (0.05/4). Two separate MANOVAs by Gender on (1) pretest locomotor and object control standard scores and (2) pretest HTKS raw scores, Go/No-Go scores, and Card Sorting scores revealed non-significant main effects for Gender (*p* > 0.05) for both analyses. Two separate MANOVAs by Session on (1) pretest locomotor and object control standard scores and (2) pretest HTKS raw scores, Go/No-Go scores, and Card Sorting scores revealed non-significant main effects for Session (*p* > 0.05) for both analyses. Overall, there were no significant differences between children who participated in full-day versus half-day Summer Success programs, nor were there differences among boys versus girls, on FMS and measures of EF. Two separate ANOVA by Gender on (1) jump distance and (2) PMC also revealed non-significant main effects for Gender (*p* > 0.05). Two separate ANOVAs by Session on (1) jump distance and (2) PMC also revealed non-significant main effects for Session (*p* > 0.05). Overall, there were no differences in FMS and EF by Session (see Table 2). Thus, in examining the impact of the SS-SKIP intervention, it was decided to only use Gender in the analyses. At baseline, most children demonstrated delays (below the 30th percentile) in FMS [38], with 71% showing delays in object control skills and 90% in locomotor skills (Figure 1).

### 3.3. Influence of SS-SKIP on FMS

Two separate ANOVAs with repeated measures by Gender on (1) locomotor SSs and (2) object control SSs were conducted to compare baseline and post-test scores for locomotor and object control SSs (Table 3). There was a significant main effect for Time for locomotor SSs with a large size (*F*[1,16] = 16.89, *p* < 0.001, η^2^ = 0.51) and a non-significant interaction (*p* < 0.05). There was also a significant Time effect for object control SSs with a large effect size (*F*[1,16] = 5.11, *p* < 0.001, η^2^ = 0.24) and a non-significant interaction (*p* < 0.05). These data suggest that locomotor (*M* = 6.19 to *M* = 8.11) and object control (*M* = 6.48 to *M* = 8.50) SSs significantly improved from pretest to post-test, indicating that the SS-SKIP intervention was effective. An ANOVA with repeated measures by Gender on jump distance revealed a non-significant main effect for Time and the interaction (*p* > 0.05).

### 3.4. Influence of SS-SKIP on PMC and EF

An ANOVA with repeated measures by Gender on PMC revealed a non-significant main effect for Time and the interaction (*p* > 0.05). PMC did not change across the SS-SKIP intervention. Three (HTKS, Go/No-Go, Card Sort) separate ANOVAs with repeated measures by Gender revealed non-significant (*p* > 0.05) Time main effects and interaction effects for Go/No-Go and the Card Sort tasks. However, there was a significant Time main effect with a large effect size (*F*[1,16] = 18.75, *p* < 0.001, η^2^ = 0.54) for HTKS but no significant interaction (*p* > 0.05). HTKS task scores improved from pretest (*M* = 25.50, *SD* = 14.48) to post-test (*M* = 31.83, *SD* = 15.99).

## 4. Discussion

The overall purpose of this pilot study was to evaluate if a 4-week pilot SS-SKIP FMS intervention delivered as part of the overall Summer Success school readiness program would significantly improve FMS in preschool-aged children about to enter kindergarten. It was hypothesized that children who participated in the SS-SKIP program would significantly improve their FMS from pretest to post-test. Additionally, a secondary research question investigated gender differences in pretest scores and whether participation in SS-SKIP would lead to significant improvements in the secondary outcomes of PMC and EF.

### 4.1. Baseline Measures of FMS, PMC, and EF

Analysis of baseline measures of FMS, PMC, long jump, and EF (HTKS, Go/No-Go, Card Sort) revealed that there were no significant differences by Gender or by Session (full-time or half-time status). Thus, it appears the children were drawn from similar backgrounds and had similar FMS, PMC skills, and EF skills prior to the intervention. An interesting finding is that there were no gender differences in object control skills, as the literature has routinely found that boys out-perform girls in these skills [6]. It may be that object control skill percentiles were so low that variation in skills was hard to find. The mean locomotor percentile was 14.62%, and the mean object control percentile was 19.19%, revealing that children were developmentally delayed in their FMS.

Not only were mean locomotor and object control skill percentiles low, but the majority of the children in this study presented FMS delays in their object control (71%) and locomotor control skills (90%) at baseline assessment. Since these children had little-to-no prior experience with formal early childhood education, it is no surprise but also concerning that their performance at baseline was very low in both domains. However, these findings are congruent with the literature that shows children in underserved populations demonstrate FMS delays due to a variety of environmental constraints [44]. In the United States, approximately 77% of preschoolers are now reported to have delays in their FMS, with many experts calling this finding an emerging epidemic [7]. Addressing FMS development in early childhood has become increasingly crucial, and the need for early intervention is apparent for children from disadvantaged backgrounds.

### 4.2. Influence of SS-SKIP on FMS

It is well documented that a variety of SKIP interventions are highly effective in improving FMS for young children from disadvantaged communities [36,44,45]. However, before this pilot study, no other SKIP program had examined the effectiveness of a short, 4-week program housed in an academic success program conducted over the summer for children that came from disadvantaged communities. A key element of Summer Success and SS-SKIP was that they served a unique group of children who had previously not experienced early childcare and thus were at greater risk of academic issues going into kindergarten. The overall goal of SS-SKIP was to remediate any potential delays in FMS, PMC, and EF prior to the start of kindergarten.

The hypothesis that children who participated in the SS-SKIP program would significantly improve their FMS was supported. As previous interventions demonstrated FMS improvements in as little as 5 weeks [46] and 6 weeks [27], this study found that a brief 4-week SS-SKIP intervention could also result in significant improvements in object control and locomotor standard scores. For children who lack access to formal schooling or daycare before starting kindergarten, this short intensive intervention could be a viable option to help bridge the gap in motor delays. The timing of this particular study was also unique, as many FMS interventions take place during the school year within childcare centers. This particular program took place in the summer and demonstrated the ability to give children a “jumpstart” in improving object control skills, which might have long-term health implications, as longitudinal research has shown that early object control skill proficiency predicts PA levels and fitness levels in older children [9,47]. This study is also unique in that the population of children who had not been previously served by early childhood centers is unique and understudied. Future research should examine the necessary conditions needed (e.g., space, equipment, teacher training) in order for SKIP to be implemented in a variety of settings.

This study supports previous findings indicating that FMS interventions effectively enhance FMS in children [16,48,49]. Specifically, SKIP has proven to be effective when delivered by Head Start teachers who received training in improving object control skills and by experts in the field of motor development [50]. Future research can examine if a 4-week SKIP intervention led by teachers or parents would bring about similar improvements. Initial findings indicate that the effectiveness of the SKIP program depends on the implementer’s experience and instructional approach [50]. The SS-SKIP intervention offered a sequence of developmentally appropriate activities for the children, focusing on practicing and reinforcing their FMS. Even though the intervention focused primarily on object control skills instruction, the children also increased their locomotor scores from pre- to post-intervention (Table 3). Notably, locomotor skills tend to emerge earlier and are generally acquired more rapidly than object control skills, which may explain the observed improvements, as locomotor skills were included in the warmup activities [5,11].

The effect sizes for the SS-SKIP were η^2^ = 0.24 for object control skills and η^2^ = 0.51 for locomotor skills, both of which indicate large effects. Previous studies on SKIP have reported effect sizes ranging from 0.55 to 0.70 [5]. While it is somewhat unexpected that a shorter duration of SKIP yielded similar effect sizes, it is encouraging that this brief version of SS-SKIP still led to significant improvements in both object control and locomotor skills. It is also not surprising that object control skills had smaller effect sizes than locomotor skills. Object control skills emerge later in the developmental repertoire and consist of complex movements that require multi-limb coordination and advanced temporal abilities. These findings warrant further investigation, as this intervention’s dosage is one of the lowest reported. Future research needs to examine the dose–response relationship and the long-term impact of SKIP via repeated retention tests to determine whether the effects are sustained over time. Future research also needs to trach the children longitudinally as they go on to childhood and adolescence to determine if an early boost in FMS is sustained over time and if it helps children remain physically active.

### 4.3. Influence of SS-SKIP on PMC and EF

An exploratory component of this study was to examine the effects the intervention had on a child’s PMC and EF. As this SS-SKIP intervention was short in duration (i.e., only one month) this may have played a factor in our findings, with PMC not showing any significant changes. It may be that the dose was not long enough for the children to have perceived their FMS as improving and correspondingly had a downstream impact on PMC. Although the findings for PMC were not significant, it may be that changes in PMC will show up later and become more pronounced over time. Designing interventions in early childhood that foster the development of FMS and PMC is important in evaluating children’s self-perceptions [51]. PMC plays a key mediating role in promoting both PA and FMS development during early childhood [19].

Another hypothesis of this study was whether the SS-SKIP intervention would bring about changes in EF, as a previous study found significant improvements in FMS and EF among disadvantaged preschoolers [45]. Consistent with prior findings [45], significant changes in the HTKS task were found after the 4-week intervention, meaning participants demonstrated better inhibitory control, attention, and cognitive flexibility following the intervention. It should be noted that other components of the Summer Success program may have contributed to these results, as prior research has linked academic success with improvements in executive function [52,53,54,55].

By contrast, no significant changes were observed in the Go/No-Go EYT or Sorting Card EYT tasks. The lack of improvement in Go/No-Go performance could indicate that the intervention may not have been as effective in enhancing sustained attention or response inhibition in this specific task. Similarly, the Sorting Card EYT resulted in no significant change from pretest to post-test, suggesting that cognitive flexibility, as assessed by the sorting task, did not experience improvement. The absence of significant changes in these tasks might be attributed to various factors, such as the nature of the tasks, individual variability in task performance, or differences in the sensitivity of these measures in capturing intervention effects. Also, these tests were administered through an iPad and, unlike the HTKS test, did not involve a lot of interaction between the tester and the child. These mixed results shed light on the complexity of measuring cognitive skills, while also demonstrating that FMS interventions can effectively enhance cognitive development alongside FMS development. The positive HTKS findings highlight the potential of SS-SKIP to enhance certain aspects of EF, but the absence of similar improvements in other EF measures suggests that interventions may need to be tailored more specifically to target a broader range of cognitive skills. This is particularly important given the growing body of evidence suggesting that FMS and EF may develop together and influence one another in a reciprocal manner [33,53,54]. Future research should continue to investigate ways to modify interventions to produce more comprehensive benefits across different tasks and domains of EF [35,56]. The results underscore the need for tailored, multifaceted interventions that address both cognitive and motor domains to optimize developmental outcomes for children in disadvantaged settings [45].

### 4.4. Limitations

This pilot study was an initial proof of concept; thus, it had several limitations. A key limitation of this study is the small sample size, which restricts the generalizability of the findings. The short, four-week intervention period may not have been long enough to capture the full extent of developmental changes in PMC and certain EF measures, potentially underestimating the intervention’s long-term impact. Furthermore, without a retention test, it is unclear whether the gains in skills were sustained beyond the immediate post-intervention period. Lastly, the absence of a control group makes it difficult to definitively attribute the observed improvements solely to the SS-SKIP intervention.

## 5. Conclusions

Children from disadvantaged environments demonstrated significant delays in their FMS at baseline, but these delays lessened because of the short intensive SS-SKIP program. This pilot study provides initial evidence that a 4-week intervention is a highly effective strategy for significantly enhancing FMS development in preschoolers within a short amount of time. As our participants had no prior school experience, the SS-SKIP intervention played a crucial role in improving their FMS competence, along with various aspects of EF, setting children on a better trajectory for school readiness.

The practical implications from this study warrant further investigation into the effectiveness of this 4-week intervention. The findings underscore a critical period in early childhood for developing FMS and EF, and practitioners should be provided with this information. The developmental delays in FMS below the 30th percentile qualifies these children for adaptive physical education services, and kindergarten testing should include a more comprehensive assessment of FMS development. Additionally, a retention test can provide more clarity on how these significant findings persist over time. Overall, the findings of this study have implications for practitioners and early childhood educators to make sure that preschool-aged children are not entering into kindergarten with these developmental delays.

## Figures and Tables

**Table 1:** Demographic statistics.

Variable	N	Mean (SD), %
**Gender**		
Boys	10	47.6%
Girls	11	
Age, Months	21	62.62(4.42)
BMI	21	15.24(2.87)
BMI %	21	47.05(36.10)
**Session**		
Session 1 (Full Day)	11	52.4%
Session 2 (Half Day)	10	47.6%
**Race/Ethnicity**		
Black/African American	13	61.9%
Multiracial	4	19.0%
Hispanic	3	14.3%
White	1	4.8%

**Table 2:** Pretest descriptives by Session.

Variables	Mean (SD)	
	Pre-Test	
	Session 1	Session 2
Locomotor SS	6.45(1.29)	5.90(2.81)
OC SS	5.82(2.52)	7.20(2.89)
PMC	3.47(0.29)	3.38(0.42)
HTKS	28.77(15.14)	23.00(13.92)
Go/No-Go	0.56(0.12)	0.46(0.17)
Sort Cards	6.33(2.59)	6.44(3.24)
Jump Distance	0.75(0.22)	0.74(0.31)

Note. Session 1 = half day; Session 2 = full day; OC = object control skills; jump distance was measured in meters.

**Table 3:** Pre/Post descriptives by sex.

Variables	Mean (SD)					
	Pre-Test			Post-Test		
	Overall	Boys	Girls	Overall	Boys	Girls
Locomotor SS *	6.19(2.11)	6.20(1.75)	6.18(2.48)	8.11(1.71)	7.88(2.03)	8.30(1.49)
OC SS *	6.48(2.73)	5.18(2.52)	7.20(2.89)	8.50(3.11)	8.25(3.01)	8.70(3.34)
PMC	3.46(0.35)	3.54(0.49)	3.40(0.21)	3.41(0.37)	3.39(0.45)	3.42(0.32)
HTKS *	25.50(14.48)	28.38(10.03)	23.20(17.45)	31.83(15.99)	34.88(13.84)	29.40(17.86)
Go/No-Go	0.51(0.15)	0.49(0.15)	0.53(0.16)	0.50(0.12)	0.49(0.13)	0.40(0.13)
Sort Cards	5.92(3.01)	5.25(3.20)	6.22(3.07)	6.31(2.75)	6.25(3.59)	6.33(2.55)
Jump Distance	0.75(0.26)	0.84(0.28)	0.65(0.20)	0.73(0.28)	0.79(0.17)	0.67(0.17)

Note. OC = object control skills. * indicates significant results (*p* < 0.001).

## Data Availability

The original contributions presented in the study are included in the article, further inquiries can be directed to the corresponding author.

## Abbreviations

The following abbreviations are used in this manuscript: fundamental motor skills (FMS), perceived motor competence (PMC), physical activity (PA), and executive function (EF).
